# A timed epigenetic switch balances T and ILC lineage proportions in the thymus

**DOI:** 10.1242/dev.203016

**Published:** 2024-12-10

**Authors:** Nicholas A. Pease, Kathryn M. Denecke, Lihua Chen, Peter Habib Gerges, Hao Yuan Kueh

**Affiliations:** ^1^ Department of Bioengineering, University of Washington, Seattle, WA 98105, USA; ^2^ Institute for Stem Cell and Regenerative Medicine, University of Washington, Seattle, WA 98105, USA; ^3^ Department of Immunology, University of Pittsburgh, Pittsburgh, PA 15213, USA

**Keywords:** Developmental timing, Epigenetic switches, Gene regulatory networks, Innate lymphoid cells, T cells, PLZF, Mouse, Bcl11b

## Abstract

How multipotent progenitors give rise to multiple cell types in defined numbers is a central question in developmental biology. Epigenetic switches, acting at single gene loci, can generate extended delays in the activation of lineage-specifying genes and impact lineage decisions and cell type output. Here, we analyzed a timed epigenetic switch controlling expression of mouse Bcl11b, a transcription factor that drives T-cell commitment, but only after a multi-day delay. To investigate roles for this delay in controlling lineage decision making, we analyzed progenitors with a deletion in a distal *Bcl11b* enhancer, which extends this delay by ∼3 days. Strikingly, delaying *Bcl11b* activation reduces T-cell output but enhances innate lymphoid cell (ILC) generation in the thymus by redirecting uncommitted progenitors to the ILC lineages. Mechanistically, delaying *Bcl11b* activation promoted ILC redirection by enabling upregulation of the ILC-specifying transcription factor PLZF. Despite the upregulation of PLZF, committed ILC progenitors could subsequently express *Bcl11b*, which is also needed for type 2 ILC differentiation. These results show that epigenetic switches can control the activation timing and order of lineage-specifying genes to modulate cell type numbers and proportions.

## Teaser

**Highlighted Article:** Delayed activation of the T-cell lineage commitment regulator *Bcl11b* in mouse thymic progenitors due to distal enhancer deletion reduces T-cell output and increases Zbtb16-driven ILC generation.

## INTRODUCTION

During development and tissue regeneration, stem and progenitor cells give rise to differentiated cell types in defined numbers and proportions to properly generate and maintain organs and body plans. To do so, these cells must closely control their lineage decisions in space and time inside an embryo or organism. The timing of lineage decisions, in particular, is subject to close developmental control, as it shapes both the degree to which progenitors can proliferate, as well as their internal regulatory state and lineage potential.

Epigenetic switching mechanisms, which modulate the activation timing of lineage-specifying genes, can influence stem cell lineage decision making (Abadie et al., 2019; Ebisuya and Briscoe, 2018). The activation timing of genes is often assumed to depend solely on the activity of upstream *trans*-factors. However, across a range of systems, epigenetic switches, acting in *cis* at individual gene loci, can delay the activation of lineage-specifying genes after initial exposure to developmental signals (Abadie et al., 2024; Berry et al., 2017; Bintu et al., 2016; Hathaway et al., 2012; Ng et al., 2018), sometimes by multiple days and cell generations. These time delays in epigenetic switching vary both between single cells and between individual gene loci in the same cell due to the stochastic nature of chromatin regulation (Bintu et al., 2016; Dodd et al., 2007; Festenstein et al., 1996; Owen et al., 2023). However, despite this stochasticity, probabilistic time constants of switching are precisely modulated at multiple levels, including by cytokine signaling, transcription factors (TFs), and associated *cis*-regulatory elements and chromatin-modifying enzymes (Chu et al., 2021; Deschamps and Duboule, 2017; Fabre et al., 2015; Kissiov et al., 2022; Pease et al., 2021). Because of their tunable nature, such timed epigenetic switches could be utilized by stem cells to control differentiation output. However, it has remained unclear what roles, if any, timing delays in epigenetic switching play in controlling stem cell lineage decisions and output.

Here, we study the functional roles for epigenetic timing delays in the lineage decisions of early thymic progenitors. After entering the thymus, hematopoietic progenitors maintain a multipotent state for ∼7 days, where they are able to give rise to multiple immune cell lineages. The large majority of progenitors then commit to the T-cell lineage, and proceed to undergo T-cell receptor rearrangement and selection to become a functional T cell. However, a fraction of progenitors can give rise to type 2 innate lymphoid cells (ILC2s) as an alternative lineage option, both in the fetal and in the adult thymus (Ferreira et al., 2021; Jones et al., 2018), or generate distinct subtypes of natural killer (NK) cells (Vargas et al., 2011; Vosshenrich et al., 2006).

The extended time period in which progenitors maintain a multipotent state is due in part to an epigenetic switch that generates a multi-day delay in the activation of the T-cell lineage commitment regulator *Bcl11b*. T-cell lineage specification begins with engagement of Notch/Delta signaling (Chen et al., 2019; Maillard et al., 2005; Radtke et al., 2010), which upregulates the expression of the TFs TCF-1 (also known as TCF7) and Gata3. These TFs, in conjunction with Runx factors that are already expressed prior to thymic entry, then work together to turn on Bcl11b expression, which represses myeloid and NK cell potential and drives T-cell commitment (García-Ojeda et al., 2013; Germar et al., 2011; Kueh et al., 2016; Shin et al., 2023; Weber et al., 2011). Strikingly, whereas TCF-1 and Gata3 turn on shortly after thymic entry and exposure to Notch ligands (∼1 day), *Bcl11b* activation occurs only ∼7 days later (Rothenberg, 2019; Yui and Rothenberg, 2014). By analyzing *Bcl11b* activation dynamics at the single chromosomal allele/single-cell level, we found that this multi-day delay was due to a timed epigenetic switch, involving a rate-limiting transition of the gene locus from a compacted, silent state to a de-compacted, expressing state (Ng et al., 2018; Pease et al., 2021). Importantly, although this switch is stochastic, time constants for probabilistic switching are tightly controlled by histone-modifying enzymes [Polycomb repressive complex (PRC)2] (Pease et al., 2021), upstream TFs (TCF-1, Gata3) (Kueh et al., 2016), as well as a far-distal *cis*-regulatory element (Li et al., 2013; Ng et al., 2018), which we termed a ‘timing enhancer’ (TE) as it moderately speeds up the onset of *Bcl11b* activation in progenitors but is not required for maintaining its expression (Chu et al., 2021).

Time-delayed control of *Bcl11b* epigenetic switching and activation may play a role in T-cell and ILC2-lineage decision making in early progenitors. Although ILCs share common transcriptional programs with T cells, they also express Id2, PLZF (encoded by the *Zbtb16* gene) and RORα, which work together to suppress T-cell programs (Qian et al., 2019; Wang et al., 2017) and to specify ILC identity (Constantinides et al., 2014; Fang and Zhu, 2017; Ferreira et al., 2021; Rothenberg, 2019). These ILC-specific regulators are prevented from being expressed in committed T-cell progenitors by expression of Bcl11b (Hosokawa et al., 2018; Zhou et al., 2022); however, because these regulators are downstream of Notch signaling and its target Gata3 (Ferreira et al., 2021; Rothenberg, 2019), they could potentially become induced in multipotent progenitors, particularly in cells that show lengthened delays in *Bcl11b* activation. Paradoxically, although Bcl11b represses *Id2* and *Zbtb16*, its expression is also required for development of ILC2s, where it binds and regulates a set of genes distinct from its target genes in T cells (Hosokawa et al., 2020). How the same TF that represses alternate-fate regulators to uphold cell identity in one lineage can also work alongside the same regulators to establish another identity in another lineage is currently unknown.

In this study, we sought to understand how developmental timing delays set by the *cis*-epigenetic switch for *Bcl11b* activation regulate T and ILC decisions in early double-negative (DN) progenitors (i.e. progenitors lacking CD4 and CD8). To disentangle *cis*-epigenetic timing control from other developmental mechanisms, we utilized a knockout mouse strain carrying a deletion of the *Bcl11b* TE (Li et al., 2013; Ng et al., 2018) (ΔTE). The TE serves as one of two transcription start sites for a distal long non-coding RNA transcript (*ThymoD*), which is activated immediately preceding *Bcl11b* activation and physically interacts with its promoter through DNA-looping mechanisms (Isoda et al., 2017). While completely disrupting *ThymoD* transcription impairs *Bcl11b* expression levels and leads to impaired T-cell development and consequently leukemia, removal of the TE only moderately delays *Bcl11b* activation by ∼3 days without affecting its maintenance and subsequent T-cell maturation and function once expressed. By analyzing lineage decision making in cells having both normal and protracted time delays in *Bcl11b* activation in the thymus and *in vitro* (Holmes and Zuniga-Pflucker, 2009), we found that delayed *Bcl11b* activation and T-cell lineage commitment reduced T-cell output, but enhanced the generation of ILC2s in the thymus. This lengthened time in an uncommitted state enabled progenitors to prime an early pro-ILC transcriptional program, marked by heightened expression of *Zbtb16*, which drove commitment down the ILC2 pathway. Importantly, progenitors were still able to activate *Bcl11b* after the onset of ILC priming; however, when *Bcl11b* was activated after priming, it no longer repressed *Zbtb16* and other ILC2 regulators, which were now stably co-expressed alongside *Bcl11b* to enable the commitment to the ILC2 fate. These results show that *cis*-epigenetic switches, by setting the relative timing of gene regulatory events, can control lineage decisions at a developmental branch point and thereby control the numbers and proportions of differentiated cells generated. More generally, our findings highlight the importance of temporal regulation in gene regulatory networks for decision making in development and tissue regeneration.

## RESULTS

### A distal enhancer modulates the activation timing of *Bcl11b* but not maintenance of its expression

It is difficult to perturb the timing of a single epigenetic switching event and lineage commitment step in isolation from other molecular processes in the cell. Knocking out an essential lineage-specifying gene would generally completely abrogate development, whereas perturbing upstream signals or *trans*-acting factors that regulate the gene of interest would lead to systemic regulatory effects that are difficult to disentangle from effects on target gene activation itself.

Mutating a *cis*-regulatory element that selectively modulates the kinetics of a gene activation event *in cis* provides a method for probing functional roles for timing in lineage decision making. From work in diverse developmental and differentiation systems, it is now established that some *cis*-acting regulatory elements specifically enhance the probability a single gene stochastically switches on in response to signals, without affecting its final expression magnitude once active. As switching probabilities are low, they can frequently give rise to extended gene activation delays that span multiple cell generations, and thus can be referred to as ‘timing enhancers’ (TEs) (Chu et al., 2021). Mutations at these TEs could provide a unique tool to investigate roles for gene activation timing (Nguyen et al., 2021), as they frequently result in moderate changes in the timing delays for gene activation and developmental events (Gérard et al., 1997; Zákány et al., 1997).

**Fig. 1.**
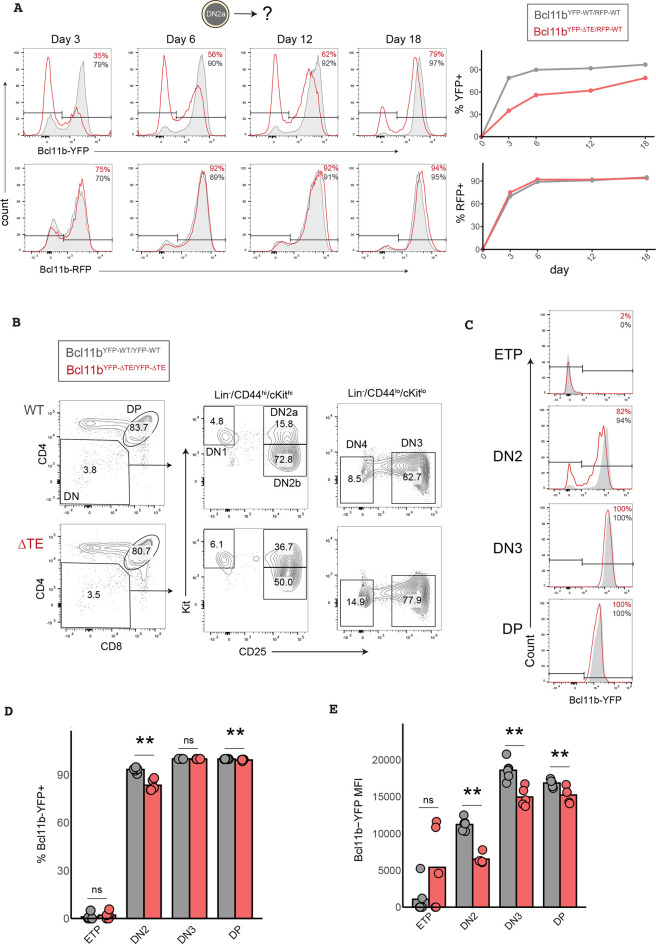
**Deletion of a distal enhancer selectively delays the timing of *Bcl11b* activation without altering its expression magnitude or maintenance.** (A) Bone marrow-derived Bcl11b-negative DN2a progenitors were sorted and re-cultured on OP9-DL1 stromal cells for up to 18 days. Graphs show flow cytometry analysis of Bcl11b-YFP and Bcl11b-RFP allele expression levels with histograms (left) and frequency of live DN progenitors positive for each allele over time (right). (B) Gating strategy for analyzing *in vivo* thymocytes. (C) Representative histograms of Bcl11b-YFP expression levels for each T-cell developmental stage. (D) Quantification of the frequency of Bcl11b-YFP^+^ thymocytes (unpaired Wilcoxon rank sum test, one-tailed, **P<0.01, *n*=6 WT mice and *n*=5 ΔTE mice). (E) Quantification of Bcl11b-YFP expression levels in thymocytes [geometric mean fluorescence intensity; unpaired Wilcoxon rank sum test (one-tailed), **P<0.01, *n*=6 WT mice and *n*=5 ΔTE mice]. MFI, median fluorescence intensity; ns, not significant.

To study the role of *Bcl11b* activation timing in regulating thymocyte differentiation, we utilized a mouse in which the *Bcl11b* TE was removed from both copies of *Bcl11b*, which were also tagged with a mCitrine yellow fluorescent protein knocked into its 3′-untranslated region (Bcl11b^YFP-ΔTE^) (Ng et al., 2018). We had previously verified that this knock-in 3′UTR reporter closely captures expression dynamics of endogenous *Bcl11b* transcripts, and does not lead to disruption in expression (Kueh et al., 2016). To first assess the effects of TE deletion on *Bcl11b* activation dynamics, we crossed this Bcl11b^YFP-ΔTE^ mouse strain to a second strain in which each wild-type copy of *Bcl11b* was non-disruptively tagged with a mCherry red fluorescent protein (Bcl11b^RFP-WT^). We purified bone marrow derived Bcl11b-negative DN2a progenitors from either Bcl11b^YFP-WT/RFP-WT^ or Bcl11b^YFP-ΔTE/RFP-WT^ mice and co-cultured them with OP9-DL1 stromal cells, which enable the reconstitution of early thymopoiesis *in vitro*. In agreement with our previous results (Ng et al., 2018), the mutant Bcl11b^YFP-ΔTE^ allele was expressed in an all-or-none manner ∼3 days later than the wild-type Bcl11b^RFP-WT^ allele in the same cells (Fig. 1A, Fig. S1A). Similarly, Bcl11b^YFP-ΔTE^ DN2a progenitors exhibited a delayed onset of YFP expression relative to Bcl11b^YFP-WT^ DN2a progenitors; however, Bcl11b^YFP-ΔTE^ DN2a progenitors were able to maintain relatively normal Bcl11b-YFP expression levels after activation (Fig. 1A). To determine whether this TE mutation could be used to study the importance *Bcl11b* activation timing *in vivo*, we next compared Bcl11b expression across all stages of thymocyte development in Bcl11b^YFP-WT/YFP-WT^ (WT) or Bcl11b^YFP-ΔTE/YFP-ΔTE^ mice (ΔTE) (Fig. 1B-E). ΔTE mice exhibited a reduction in the percentage of Bcl11b-YFP^+^ DN2 progenitors, confirming that Bcl11b activation is delayed at the DN2 stage *in vivo* (Fig. 1D). However, as observed *in vitro*, ΔTE thymocytes at the DN3-DP stages maintained comparable levels of Bcl11b expression post-activation and thymocyte maturation (Fig. 1C,E). Together, these results demonstrate that removal of the *Bcl11b* TE selectively delays *Bcl11b* activation at the DN2 stage without disrupting *Bcl11b* expression levels and subsequent stages of T-cell development.

### Delayed *Bcl11b* activation decreases T-cell output and increases thymic ILC output

**Fig. 2.**
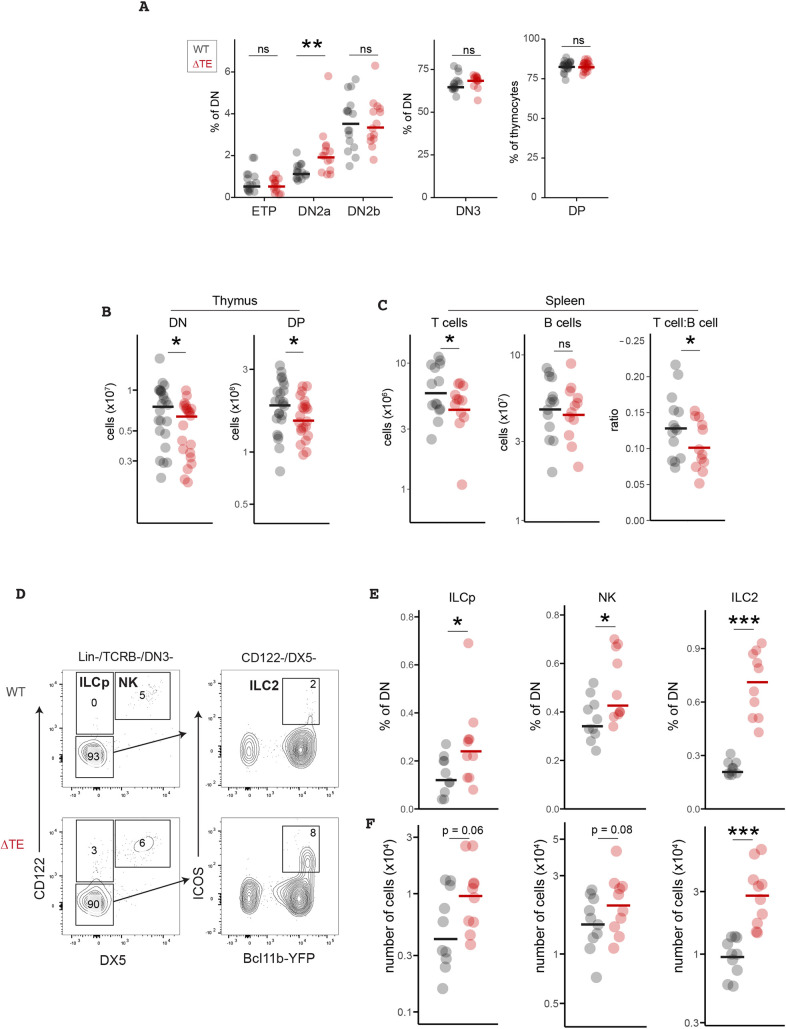
**Delaying *Bcl11b* activation enhances ILC generation while reducing T-cell output.** (A) Quantification of thymocyte population frequencies and total cell numbers (unpaired Wilcoxon rank sum test, two-tailed, **P<0.01, *n*=16 WT separate mice and *n*=14 ΔTE separate mice). (B) Quantification of DN or DP thymocyte numbers (unpaired Wilcoxon rank sum test, one-tailed, *P<0.05, *n*=16 individual WT mice and *n*=14 individual ΔTE mice for DN thymocytes, *n*=24 separate WT mice and *n*=22 separate ΔTE mice for DP thymocytes). (C) Quantification of splenocyte T- and B-cell numbers (unpaired *t*-test, one-tailed, *P<0.05, *n*=16 separate WT mice and *n*=14 separate ΔTE mice). (D) Gating strategy for analyzing thymic ILC subsets. (E,F) Quantification of thymic ILC subset frequencies and total cell numbers (unpaired Wilcoxon rank sum test, one-tailed, *P<0.05, ***P<0.001, *n*=10 separate mice per genotype). ns, not significant.

To investigate how delayed *Bcl11b* activation timing may impact T-cell development *in vivo*, we compared frequencies and numbers of DN thymocytes at different stages in the thymi of WT and ΔTE mice. We found that ΔTE mice showed significantly increased percentages of DN2a progenitors (∼1.7-fold), consistent with the delayed *Bcl11b* activation impeding the transition of these progenitors to the T-cell-committed DN2b stage in the thymus (Fig. 2A). This increase in DN2a frequency in ΔTE mice did not translate into significantly elevated cell numbers (Fig. S2A), suggesting that progenitors do not simply arrest and accumulate at this pre-commitment stage, but may become redirected into an alternate developmental pathway. All downstream subsets [DN2b, DN3 and CD4^+^CD8^+^ double positive (DP)] had similar frequencies between WT and ΔTE mice, indicating that delaying *Bcl11b* does not disrupt T-cell development and maturation after lineage commitment (Fig. 2A). However, ΔTE mice did show mild but statistically significant decreases in their total numbers of DN thymocytes (15% decrease), DP thymocytes (19% decrease) (Fig. 2B), as well as peripheral T cells in the spleen (27% decrease for total splenic T cells and 20% decrease in splenic T:B cell ratio) among a large number of matched mice (*n*=14-16 mice per genotype for DN counts and *n*=22-24 per genotype for DP counts). (Fig. 2C). Importantly, the number of B cells in the spleen remained constant (Fig. 2C), suggesting that these changes in T-cell numbers are not due to systemic changes in immune cell development in ΔTE mice. These results indicate that a delayed *Bcl11b* activation and T-lineage commitment due to TE deletion leads to a mild attenuation in T-cell output from the thymus.

Activation of *Bcl11b* is important for silencing of ILC lineage-specifying genes in DN2 progenitors (Hosokawa et al., 2020; Kueh et al., 2016) and its deletion results in the acquisition of an immature ILC-like state and increased NK cell production (Hosokawa et al., 2018; Li et al., 2010; Zhou et al., 2022). Therefore, we hypothesized that delaying *Bcl11b* activation diverts DN2a progenitors away from the T-cell lineage and towards the ILC pathway in the thymus. To investigate this hypothesis, we quantified the frequencies and numbers of ILC precursors (ILCp) [Lin^−^/TCRB^−^/CD122 (IL-2Rβ)^+^/DX5 (integrin α2)^−^], ILC2s (Lin^−^/TCRB^−^/CD122^−^/DX5^−^/ICOS^+^/Bcl11b^+^) and NK cells (Lin^−^/TCRB^−^/CD122^+^/DX5^+^) in thymi of WT and ΔTE mice (Fig. 2D-F). Indeed, compared to the WT mice, ΔTE mice exhibited significantly elevated frequencies and numbers of ILCp and ILC2 cells in the thymus, and also exhibited moderate increases in the frequencies of NK cells. These cell populations largely exited the DN2a compartment and adopted an apparent DN4 phenotype with low CD25 (IL-2Rα) and Kit levels (Fig. S2B,C). However, a small fraction of ILCp from ΔTE mice retained a DN2a phenotype with elevated CD25 and Kit levels, consistent with these cells originating from uncommitted progenitors. Together, these results indicate that a prolonged delay in the activation timing of *Bcl11b* enhances production of multiple ILC lineages. Notably, ILC2s generated from ΔTE progenitors expressed Bcl11b at similar levels compared to those generated from WT progenitors. This similarity indicates that differences in ILC2 numbers between WT and ΔTE mice arise due to differences in activation timing and not dosage differences in *Bcl11b* expression. Together, these results demonstrate that the timing of *Bcl11b* activation is important for determining the relative proportions of T-cell and ILC progenitors in the thymus.

### Delayed *Bcl11b* activation promotes ILC diversion in multipotent thymic progenitors

**Fig. 3.**
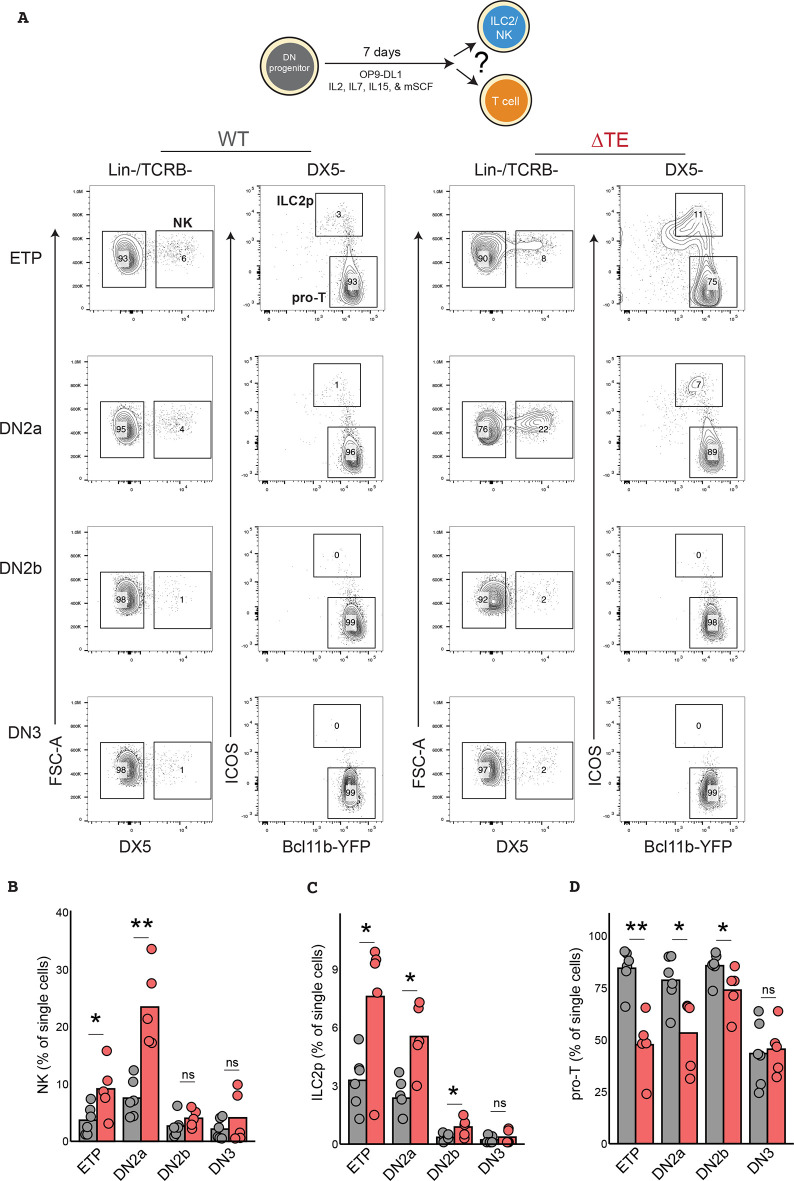
**Delayed *Bcl11b* activation promotes ILC redirection in progenitors poised for T-cell commitment.** (A) DN thymocytes were sorted and re-cultured for 7 days on OP9-DL1 stromal cells in the presence of pro-T and pro-ILC cytokines (IL-2, IL-7, IL-15, Flt3-L, SCF). (B-D) Quantification of the frequencies of NK, ILC2 and T-cell progenitors at day 7 (unpaired Wilcoxon rank sum test, one-tailed, *P<0.05, **P<0.01; *n*=5 WT and 6 ΔTE separate mice. ns, not significant.

Previous studies had shown that thymic progenitors retain the capacity to differentiate into ILCs for multiple days after thymic entry, prior to *Bcl11b* activation and T-cell lineage commitment (Qian et al., 2019; Wang et al., 2017). These observations raise the possibility that the delay in *Bcl11b* activation due to TE deletion may promote ILC diversion at multiple stages leading up to T-cell commitment by prolonging the time uncommitted progenitors spend in this multipotent state. To test this hypothesis directly, we compared the ILC lineage potential of WT versus ΔTE thymocytes at multiple stages, both before and after T-cell lineage commitment. To do so, we sorted pre-commitment [early thymic progenitors (ETPs) and DN2a] and post-commitment (DN2b and DN3) thymic progenitors from the thymi of WT and ΔTE mice, co-cultured them with OP9-DL1 stromal cells and cytokines to facilitate both ILC and T-cell development for 7 days before analyzing them by flow cytometry (Fig. 3A). As expected, ETP and DN2a progenitors from WT mice gave rise to ILC2s and NK cells at frequencies similar to those previously observed in the presence of sustained Notch signaling (Koga et al., 2018), whereas DN2b and DN3 thymocytes showed significantly decreased production of both ILC lineages, consistent with T-cell lineage commitment occurring at the DN2b stage (Fig. 3B-D). TE deletion decreased the T-cell production while increasing NK and ILC2 generation from both ETP and DN2a progenitors. ILC production was most enhanced when starting from DN2a progenitors, consistent with this lineage bifurcation occurring at this later stage when cells are poised to turn on *Bcl11b*. In contrast, DN3 progenitors from ΔTE mice generated normal frequencies of T-cell progenitors and failed to generate a significant number of ILC2 or NK cells, consistent with these progenitors being T-cell lineage restricted. Together, these results demonstrate that delaying the onset of *Bcl11b* activation increases the likelihood of DN2 progenitor divergence away from the T-cell lineage towards the ILC pathway in a thymocyte cell-intrinsic manner.

### Delayed *Bcl11b* activation leads to ILC priming and *Zbtb16* upregulation in multipotent progenitors

**Fig. 4.**
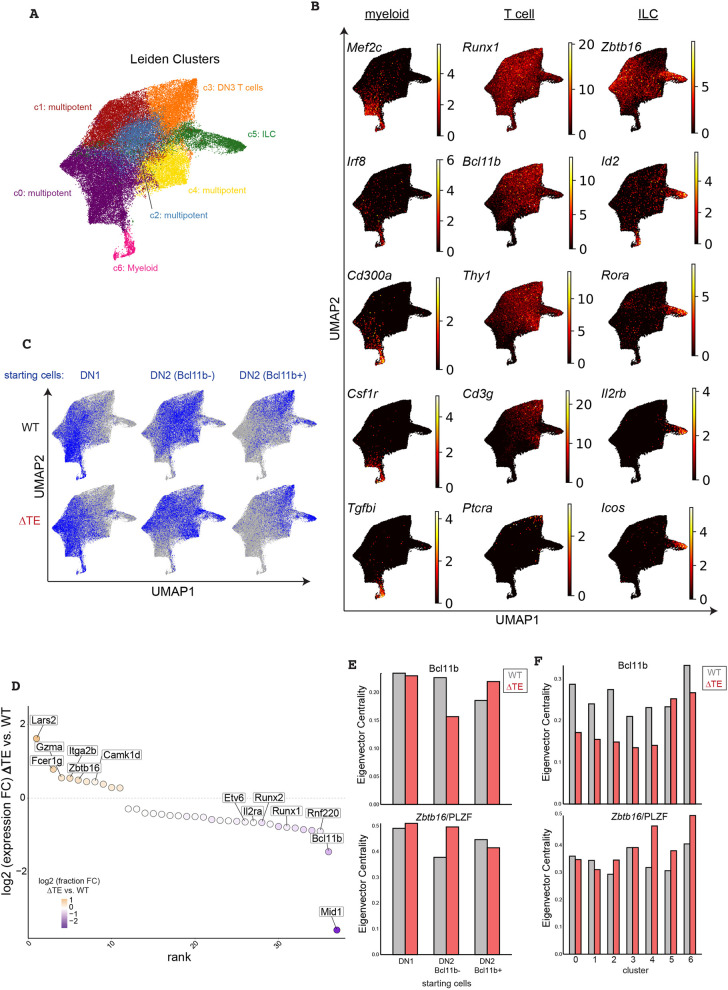
**Delayed *Bcl11b* activation enables emergence of an ILC program in poised progenitors marked by *Zbtb16* upregulation.** (A) UMAP representation of all cells with Leiden cluster labels and annotation (pooled bone marrow-derived progenitors from six to eight mice per genotype). (B) UMAP representation of all cells colored by myeloid, T-cell or ILC lineage marker gene expression levels (normalized to total counts). (C) UMAP representation of single-cell RNA-sequencing experiment colored by starting cell population for each sample (pooled bone marrow-derived progenitors from six to eight mice per genotype). (D) Differential gene expression analysis between ΔTE and WT cells in the multipotent clusters (clusters 0, 1, 2, and 4 only). Only genes detected in >5% of cells with an adjusted *P*<0.05 and an absolute fold-change >1.2 are displayed. (E,F) Eigenvector centrality values for Bcl11b and Zbtb16 in CellOracle-generated gene regulatory network models based on starting cell state (E) or Leiden cluster label (F).

The prolonged delay in *Bcl11b* activation due to TE deletion may promote ILC divergence by enabling a parallel and competing pro-ILC transcriptional program to emerge in DN2a progenitors. To test this hypothesis, we purified bone marrow-derived DN1 (CD44^+^CD25^−^), DN2a (CD44^+^CD25^+^Bcl11b^−^), and DN2b (CD44^+^CD25^+^Bcl11b^+^) progenitors from WT or ΔTE mice and re-cultured them on OP9-DL1 stromal cells for 4 days before harvesting them for single-cell RNA sequencing (Fig. 4A-C). We chose this range of initial progenitors to ensure adequate sampling of cells across different developmental states, and also to enable concurrent analysis of lineage potential for different progenitor states through sequencing. We utilized a combinatorial indexing strategy for single-cell profiling (Cao et al., 2019), which yielded high-quality transcriptomes from 68,464 cells across these six conditions (Fig. 4A-C). To clearly resolve developmental lineages from single-cell data, we used a probabilistic topic modeling approach, which represents cells according to collections of co-expressed genes (i.e. gene topics) (Lynch et al., 2022). Here, each cell is represented by a mixture of gene topics, each with unique weights according to its gene expression profile (Fig. S3A, Table S1). We then visualized cells based on their unique topic weights in two-dimensions using a uniform manifold approximation projection (UMAP), and clustered cells using the Leiden algorithm to visualize developmental states and trajectories.

This visualization revealed distinct trajectories corresponding to the progression of multipotent progenitors into the myeloid, T-cell or ILC lineages based on marker gene expression (Fig. 4B,C). Multipotent progenitors occupied the left region of the UMAP (clusters 0, 1, 2, 4) and gave rise to three lineages, myeloid progenitors (cluster 6), DN3 T cell precursors (cluster 3) as well as ILCs (cluster 5). As expected, clusters from these lineages were enriched for expression of corresponding lineage-specific genes [e.g. *Cd3g* and *Ptcra* for T cell precursors (cluster 3); *Il2rb*, *Icos* and *Rora* for ILC precursors (cluster 5); *Csf1r* and *Mef2c* for myeloid progenitors (cluster 6)]. Myeloid lineages appeared to be specified early, branching off from early progenitors (cluster 0) that can also give rise to T and ILC potent progenitors; in contrast, ILC lineages appeared to be specified later, branching off progenitors (clusters 1, 2, and 4) that already appeared to have some expression of T-cell lineage-associated genes (*Thy1*, *Bcl11b*). These results are consistent with the ILC lineage decision point occurring late, immediately prior to T-cell lineage commitment in DN2 progenitors.

Analysis of progenitor populations from WT and ΔTE mice showed enhanced ILC specification as a result of delayed *Bcl11b* activation, consistent with the results obtained from thymus-derived progenitors (Fig. 3). Starting with DN1 progenitors from WT and ΔTE mice generated some myeloid progenitors (cluster 6), but primarily gave rise to T-cell and ILC progenitors (clusters 1, 2 and 4), reflecting a transition to a T/ILC bipotent state yet to be perturbed by a forthcoming delay in *Bcl11b* activation (Fig. 4C). In contrast, starting with DN2 *Bcl11b*^−^ progenitors generated very few myeloid progenitors, but instead gave rise to T-cell or ILC precursors, reflecting their more advanced developmental state. Together, these results demonstrate that DN2 progenitors retain ILC developmental potential and that delayed *Bcl11b* activation and T-cell lineage commitment promotes divergence towards a pro-ILC transcriptional program within which late-coming Bcl11b expression can operate to promote ILC2 differentiation.

To identify the transcriptional regulators that promote ILC divergence in DN2 progenitors when *Bcl11b* activation is delayed, we analyzed genes differentially expressed between WT and ΔTE progenitors (clusters 0, 1, 2 and 4) that still harbor T and ILC lineage potential. From this analysis, we found that progenitors with a deleted enhancer showed significantly decreased expression of *Bcl11b*, as expected. ΔTE progenitors also showed reduced expression of other T-cell lineage regulators, including *Runx1* and *Runx2*, suggesting the emergence of alternate regulatory programs that begin to dampen T-cell lineage gene expression when *Bcl11b* activation is delayed (Fig. 4D). Indeed, progenitors from ΔTE mice showed heightened expression of several genes associated with ILCs or NK cells, including *Fcer1g*, *Gzma* and *Zbtb16*, which encodes the pro-ILC TF PLZF. Intriguingly, though ILC specification requires the concerted action of three regulators – *Zbtb16*, *Id2* and *Rora* (Constantinides et al., 2014; Fang and Zhu, 2017; Ferreira et al., 2021; Rothenberg, 2019) – only *Zbtb16* was noticeably upregulated in uncommitted progenitors upon *Bcl11b* enhancer deletion. In contrast, *Id2* and *Rora* both showed similarly low expression levels in multipotent progenitors with a deleted *Bcl11b* enhancer, and increased in expression only after ILC pathway entry (Fig. 4B).

These findings, along with observations that the *Zbtb16* locus is directly bound and repressed by Bcl11b during T-cell commitment (Hosokawa et al., 2018, 2020), implicate *Zbtb16*/PLZF as a driver of ILC lineage divergence at the DN2 stage when *Bcl11b* expression is delayed. To investigate whether *Zbtb16*/PLZF has elevated regulatory activity when *Bcl11b* is delayed, we used CellOracle gene regulatory network analysis to infer the centrality of *Zbtb16*/PLZF to the transcriptional programs of ΔTE and WT progenitors. CellOracle applies a linear machine-learning model to predict cluster-specific TF-to-gene linkages based on TF motif occurrence at regulatory regions associated with a target gene as well as the relationship between TF expression and that of the candidate target gene (Kamimoto et al., 2023). As expected, the CellOracle model predicted Bcl11b has reduced centrality in the network of ΔTE-derived progenitors starting from the DN2 Bcl11b^−^ state and across all multipotent clusters (0, 1, 2, 4) (Fig. 4E,F). Conversely, *Zbtb16*/PLZF had higher centrality scores in ΔTE-derived progenitors starting in the DN2 Bcl11b^−^ state compared to their WT counterparts. Specifically, the predicted *Zbtb16*/PLZF centrality is most dramatically elevated in ΔTE progenitors in cells of the multipotent clusters 2 and 4 as well as the ILC cluster 5. As expected, several of the top predicted *Zbtb16*/PLZF target genes were differentially expressed between ΔTE-derived and WT-derived DN2 Bcl11b^−^ progenitors (*Pde4d*, *Camk1d*, and *Runx1*) (Fig. S3B). Together, these findings suggest that *Zbtb16* upregulation in DN2 progenitors due to delayed *Bcl11b* activation may initiate a pro-ILC program in these cells.

**Fig. 5.**
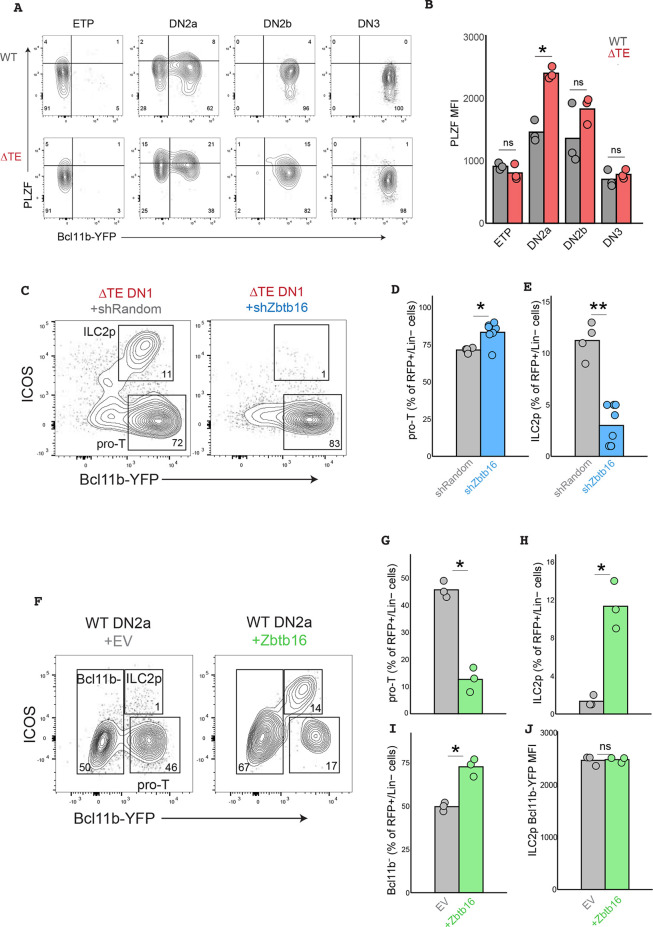
***Zbtb16*** **mediates ILC redirection in progenitors with delayed *Bcl11b* activation.** (A) Representative scatter plots of PLZF and Bcl11b-YFP levels across *in vivo* DN thymocyte populations. (B) Quantification of PLZF levels (geometric mean fluorescence intensity) in thymocytes (unpaired Wilcoxon rank sum test, *P<0.05, *n*=3 separate mice per genotype). (C) Bone marrow-derived DN1 progenitors from ΔTE mice were retrovirally transduced, sorted and re-cultured on OP9-DL1 cells in the presence of pro-ILC and pro-T cell cytokines for 10 days. (D,E) Quantification of the frequencies of pro-T and pro-ILC progenitors at day 10 [unpaired Wilcoxon rank sum test, one-tailed, *P<0.05, **P<0.01; *n*=4 (shRandom) or 8 (shZbtb16) experimental replicates using bone marrow-derived cells from a single pool of ΔTE mice]. (F) Bone marrow-derived DN2a progenitors from WT mice were retrovirally transduced, sorted and re-cultured on OP9-DL1 cells in the presence of pro-ILC and pro-T cell cytokines (IL-2, IL-7, IL-15, Flt3-L, SCF) for 4 days. (G,H) Quantification of the frequencies of pro-T and pro-ILC progenitors at day 4 [unpaired Wilcoxon rank sum test, one-tailed, *P<0.05, *n*=3 independent batches of bone marrow (6-8 mice per batch) independently transduced and cultured per condition]. (I) Quantification of the frequencies of Bcl11b-negative progenitors at day 4 [unpaired Wilcoxon rank sum test, one-tailed, *P<0.05, *n*=3 separate batches of bone marrow (6-8 WT mice per batch) per condition]. (J) Quantification of the frequencies of Bcl11b-YFP MFI among ILC2p at day 4 [unpaired Wilcoxon rank sum test, one-tailed, *P<0.05, *n*=3 separate batches of bone marrow (6-8 WT mice per batch) per condition]. ns, not significant.

To investigate whether delayed *Bcl11b* activation due to enhancer deletion also facilitates *Zbtb16*/PLZF upregulation *in vivo*, we measured PLZF protein levels in progenitors from thymi of WT and ΔTE mice using immunofluorescence staining (Fig. 5A). In wild-type thymic progenitors, PLZF was low in ETPs, and was transiently upregulated at the DN2a stage before being downregulated as Bcl11b expression increased at the DN3 stage (Fig. 5A,B), in agreement with our single-cell RNA-sequencing results on *in vitro-*derived T-cell progenitors. In progenitors from ΔTE mice, this transient expression of PLZF was elevated at the DN2a stage and persisted through the DN2b stage before again being downregulated in DN3 cells. These results indicate that the ILC priming in uncommitted DN2 progenitors, as observed in *in vitro*-generated T-cell progenitors, also occurs during T-cell development in the thymus.

### *Zbtb16* promotes ILC diversion in T-cell progenitors

The heightened levels of *Zbtb16*/PLZF in DN2 progenitors experiencing delayed Bcl11b expression may be responsible for the increased frequency of thymic progenitor divergence into the ILC pathway. To test this hypothesis, we knocked down *Zbtb16* expression in bone marrow-derived DN1 progenitors from ΔTE mice through retroviral transduction of small hairpin (sh)RNAs targeting *Zbtb16* (shZbtb16), while transducing a non-targeting random shRNA as a negative control (shRandom). We then cultured transduced progenitors on OP9-DL1 monolayers in the presence of ILC-promoting cytokines and analyzed them by flow cytometry after 10 days (Fig. 5C-E). Under these conditions, the majority of progenitors primarily activated *Bcl11b* and committed to the T-cell lineage, for both shRandom and shZbtb16b-transduced cells, with a small percentage of cells entering the ILC2 lineage (Lin^−^ICOS^+^Bcl11b^+^). Progenitors transduced with shZbtb16 constructs generated significantly more T-cell progenitors, as well as significantly fewer ILC2 progenitors than their control counterparts transduced with shRandom constructs.

The elevation in *Zbtb16*/PLZF levels prior to *Bcl11b* activation, occurring as a result of *Bcl11b* TE deletion, may be sufficient to induce ILC redirection. To test this hypothesis, we investigated whether overexpressing *Zbtb16* in progenitors before Bcl11b activation enhances divergence into the ILC lineage away from the T-cell lineage. To do so, we transduced bone marrow-derived DN progenitors with an empty vector (EV) retroviral construct or one that constitutively expresses *Zbtb16*, then used cell sorting to isolate transduced DN2 progenitors not yet expressing Bcl11b. We then cultured and sorted transduced progenitors on OP9-DL1 monolayers in the presence of ILC-promoting cytokines and analyzed them by flow cytometry after 4 days (Fig. 5F). Indeed, DN2a progenitors transduced with the *Zbtb16* overexpression construct generated a significantly lower fraction of T-cell precursors (Lin^−^ICOS^−^Bcl11b^+^, 12% versus 45%) and a significantly higher fraction of ILC2 precursors (Lin^−^ICOS^+^Bcl11b^+^, 1% versus 11%), consistent with our hypothesis (Fig. 5G,H). The fraction of progenitors activating Bcl11b was reduced by *Zbtb16* overexpression (Fig. 5F,I), suggesting that *Zbtb16*/PLZF may also promote other ILC lineages that do not express Bcl11b. However, Bcl11b-YFP levels in ICOS-expressing ILC2 precursors were similar with or without *Zbtb16* overexpression (Fig. 5J), indicating that these two regulators can be stably co-expressed in these cells to drive their differentiation. Taken together, our results demonstrate that the order in which DN2 thymic progenitors upregulate *Bcl11b* versus *Zbtb16* expression determines their fate decisions: earlier expression of *Zbtb16* before *Bcl11b* favors ILC differentiation, whereas earlier expression of *Bcl11b* instead drives T-cell lineage commitment. These results highlight the importance of the relative timing of TF expression during progenitor cell fate decisions.

## DISCUSSION

Our results underscore the importance of developmental timing delays in controlling lineage decisions at developmental branch points. For ILC2 and T-cell lineage specification from DN2 progenitors, we find that extended time delays in *Bcl11b* activation provide an opportunity for cells to divert from the canonical T-cell lineage to an alternative ILC lineage. Although ILCs are relatively rare in the adult thymus, recent evidence suggests that small numbers of resident ILC2s play a crucial role in responding to thymic damage by promoting thymic epithelial differentiation to counteract thymic involution (Nevo et al., 2024). Thus, flexible and dynamic regulation of DN2-derived ILC2 populations balanced against T-cell production in the thymus may be an important aspect of thymus functionality.

Our work suggests that delayed activation of *Bcl11b*, which is known to negatively regulate *Zbtb16* upon expression, may enable an extended period of *Zbtb16* expression prior to Bcl11b expression and result in a divergence of cells into the ILC pathway. Intriguingly, *Bcl11b* activation can still occur after upregulation of the PLZF-dependent ILC program; in this case, there is a shift in transcriptional regulatory activity of Bcl11b from an pro-T-cell program to a pro-ILC program (Hosokawa et al., 2018, 2020). In an unperturbed system, the Bcl11b switch clearly occurs far more quickly on average than that of the PLZF-mediated switch and thus it is normally rare for Bcl11b^−^/PLZF^hi^ ILC progenitors to emerge from DN2 progenitors (Figs 2D-F and 5A). Our results demonstrate that the *Bcl11b* TE is responsible for upholding the rate of *Bcl11b* switching in relation to other PLZF-related switches operating in parallel and thus explains why removal of the TE increases ILC lineage generation at the expense of T-cell output. This may also explain why others have found that under wild-type conditions a small fraction of peripheral ILC2s appear to be derived from thymic DN3 progenitors, but that fraction dramatically increases when the repressors of *Zbtb16* – E2A/HEB E proteins – are disrupted (Qian et al., 2019). Interestingly, *Zbtb16* may also be regulated by its own TE during NK T-cell development, raising the possibility that competing timed switches controlling Bcl11b and *Zbtb16* activation may jointly regulate innate and adaptive fate output in the thymus.

The stochastic nature of the time delays in epigenetic switching may enable multipotent progenitors to generate multiple differentiated cell types in response to a common signal. While Notch signaling and its downstream regulators Gata3 and TCF-1 are essential for driving T-cell lineage commitment, they also play crucial roles in ILC development and also act upstream of the ILC regulators Id2 and PLZF. Thus, it is possible that these same instructive signals for T-cell development also drive ILC development in the thymus. In this scenario, the decision for whether to enter the T or the ILC pathway would be determined by the activation timing of *Bcl11b*, and possibly of *Zbtb16*, which may be also regulated by an enhancer-mediated, stochastic epigenetic switch (Mao et al., 2017). As activation time delays would vary between cells due to the inherently stochastic nature of epigenetic switching, they could generate heterogeneity in fate outcomes to modulate relative cell population sizes, even amid uniform developmental signals.

While stochastic epigenetic timing control mechanisms are likely important for controlling differentiation outcomes in diverse contexts, they may be particularly useful for lineage control in the immune system, because immune cells are highly motile and thus less confined to specific niches with defined signaling environments compared to other cell types. We note that, despite its inherently stochastic nature, epigenetic switches can be highly controlled at multiple levels, including by TFs, *cis*-regulatory elements and chromatin-modifying enzymes (Jacobsen et al., 2017; Kueh et al., 2016; Nguyen et al., 2021; Pease et al., 2021). These multiple layers of control would allow for precise tuning of differentiated cell numbers, particularly at the population level where variability in outcomes can be minimized due to averaging at large cell numbers (Nguyen et al., 2021).

In further studies, it would be important to more broadly investigate roles for epigenetic timing control in developmental gene regulation and decision making across diverse contexts. Timed epigenetic switches, when operating in the context of gene regulatory networks, have the ability to profoundly shape their dynamics and the developmental transitions they control. An understanding of these dynamics, both at an experimental and theoretical level, will help us more fully understand the developmental specification and evolutionary diversification of metazoan tissue size, shape and function.

## MATERIALS AND METHODS

### Animal models

C57BL/6 *Bcl11b^YFP/YFP^* mice (WT) and *Bcl11b^YFP^*^ΔEnh*/YFP*ΔEnh^ mice (ΔTE) were generated as previously described (Ng et al., 2018). *Bcl11b^YFP/YFP^* were crossed to *Bcl11b^YFP^*^ΔEnh*/YFP*ΔEnh^ and the resulting *Bcl11b^YFP/YFP^*^ΔEnh^ heterozygotes were used as breeding pairs to generate WT and ΔTE littermates for primary cell analysis. All animals were bred and maintained at the University of Washington, USA. Combinations of male and female mice were used for all experiments. Bone marrow for *in vitro* cultures was harvested from mice between 2 and 4 months old. Thymocytes and splenocytes were harvested from 3-week-old mice. All animal protocols were reviewed and approved by the Institute Animal Care and Use Committee at the University of Washington (Protocol No: 4397-01).

### Cell line culture

Primary cells isolated from bone marrow were cultured on a OP9-DL1 monolayer stromal cells (Holmes and Zuniga-Pflucker, 2009) at 37°C in 5% CO_2_ conditions with standard culture medium [80% MEM-alpha (Gibco), 20% fetal bovine serum (Corning), Pen-Strep-Glutamine (Gibco)] supplemented with appropriate cytokines indicated below. Phoenix-Eco cells (ATCC, CRL-3214, RRID:CVCL_H717) were cultured at 37°C in 5% CO_2_ with standard culture medium [90% DMEM (Gibco), 10% fetal bovine serum (Corning), Pen-Strep-Glutamine (Gibco)]. All cell lines were tested and found to be negative for *Mycoplasma* contamination.

### Cell purification

Bone marrow progenitors used for *in vitro* T-cell development assays were purified as previously described using CD117 Microbeads (Miltenyi Biotec, 130-091-224) (Ng et al., 2018). For all thymus analysis, thymi from 3-week-old mice derived from heterozygous crosses were mechanically dissociated before pooling and re-suspending in Fc blocking solution with 2.4G2 hybridoma supernatant. Early stage thymocytes (ETP-DN4) and ILC populations were depleted of CD4 and CD8 thymocytes before analysis or sorting. Thymocyte suspensions were labeled with biotinylated CD4 and CD8 antibodies incubated with MACS Streptavidin Microbeads (Miltenyi Biotec, 130-048-101) in HBH buffer [Hank Balanced Salt Solution (HBSS; Gibco, 14025092), 0.5% bovine serum albumin (BSA) (Sigma-Aldrich, A3294), 10 mM HEPES, (Gibco, 15630080)], pre-filtered through a cell strainer, and passed through an LS column (Miltenyi Biotec, 130-042-401).

### *In vitro* differentiation of T-cell progenitors

To generate DN T cells *in vitro*, thawed CD117 (c-Kit)-enriched bone marrow progenitors, pooled from six to eight mice per batch, were cultured on OP9-DL1 stromal cell monolayers as described previously (Ng et al., 2018) using standard culture medium [80% αMEM (Gibco, 12571063), 20% fetal bovine serum (Corning, 35-010-CV), Pen-Strep-Glutamine (Gibco, 10378016)], grown at 37°C in 5% CO_2_ conditions. All *in vitro* T-cell generation cultures were supplemented with 5 ng/ml Flt3-L (PreproTech, 300–19) and 5 ng/ml IL-7 (PeproTech, 200–07). Experiments involving *in vitro* generation of NK and ILC populations (Figs 3, 4, and 5) were supplemented with 5 ng/ml IL-7 (PreproTech, 217-17), 10 ng/ml IL-2 (PreproTech, 212-12), 10 ng/ml SCF (human stem cell factor) (PreproTech, 300–07), 10 ng/ml IL-15 (Peprotech, 210-15).

### Flow cytometry and cell sorting

Fluorescence activated cell sorting was used to isolate DN cells of interest with the following protocol. Bone marrow-derived cell cultures were scraped and incubated in 2.4G2 Fc blocking solution and stained with anti-CD25 APC-eFluor 780 and with biotinylated antibodies against a panel of lineage markers [CD19, CD11b (Itgam), CD11c (Itgax), NK1.1 (Klrb1c), Ter119 (Ly76), CD3ε, Gr-1 (Ly6g) and B220 (Ptprc) (BioLegend)] (see Table S2). Stained cells were washed with HBH (HBSS with 0.1% BSA and 10 mM HEPES) and stained with streptavidin-PerCP/Cy5.5 (BioLegend). Stained cells were washed, resuspended in HBH, and filtered through a 40-um nylon mesh for sorting with a BD FACS Aria III (BD Biosciences) with assistance from the University of Washington Pathology Flow Cytometry Core Facility. A benchtop Attune NxT Flow Cytometer (Thermo Fisher Scientific) was used to analyze primary and re-cultured thymocytes and acquired data were analyzed with FlowJo software (Tree Star).

### Single-cell RNA sequencing

Bone marrow progenitors attached to OP9-DL1 stromal cells were washed three times with PBS before scraping in HBH buffer and passing through a 70 μM mesh filter to minimize OP9-DL1 stromal cell contamination. Cells were then washed with PBS and fixed in 4% paraformaldehyde for 15 min on ice. After fixation, cells were pelleted at 500 ***g*** for 3 min at 4°C before resuspending in 1 ml of PBSR [PBS, pH 7.4, 1% BSA, 1% SUPERase-In RNase Inhibitor (Thermo Fisher Scientific), and 1% 10 mM DTT], pelleting again at 500 ***g*** for 5 min at 4°C and resuspending in PBSR. The Brotman Baty Institute (BBI) then performed the single-cell transcriptome library construction following the sci-RNA-seq3 combinatorial indexing protocol as described by Cao et al. (2019).

### Single-cell RNA-sequencing analysis

Scanpy (Wolf et al., 2018) was used to first filter on cells with at least 200 genes and genes detected in at least 15 cells. Next, cells with greater than 2000 gene expression counts or greater than 30% mitochondrial counts were removed. MIRA topic modeling was then applied with the most highly variable genes to describe each cell's transcriptional state as a composition of co-regulated genes (i.e. topics) with variable weights (Lynch et al., 2022). The nearest neighbors distance matrix was computed with *n_neighbors* set to 40 and the Leiden algorithm was used to cluster cells with *resolution* set to 0.3. For visualization and downstream analyses, cells were downsampled such that each genotype had equal numbers of cells in each starting state (12,353 for DN1 starting cells; 13,050 for DN2a starting cells; 5631 for DN2b starting cells). Differential gene expression analysis between genotypes among the multipotent DN2 clusters (0, 1, 2, 4) was performed using the Wilcoxon rank sum test. Gene ontology analysis of the top 400 genes from the T-cell and ILC topics (Table S1) was performed with the TopFun function of the TopGene suite (Chen et al., 2007).

### Retroviral vector generation and transduction

shRNA sequences targeting *Zbtb16* transcripts were joined to a U6 promoter and cloned into a Banshee-mCherry backbone as described previously (Kueh et al., 2016). The hairpin sequence is as follows: GGAAATGATGCAGGTAGATGA(anti-sense)-TTCG(loop)-TCATCTACCTGCATCATTTCC(sense).

Retroviral particles were generated using the Phoenix-Eco packaging cell line. Viral supernatants were collected at 2 and 3 days after transfection and immediately frozen at −80°C. To infect bone marrow-derived T-cell progenitors, 33 μg/ml RetroNectin (Clontech) and 2.67 μg/ml of DL1-extracellular domain fused to human IgG1 Fc protein (a gift from I. Bernstein, Fred Hutch Cancer Center, Seattle, WA, USA) were added in a volume of 250 μl per well in 24-well tissue culture plates (Costar, Corning) and incubated overnight. Viral supernatants were added the next day into coated wells and centrifuged at 2000 ***g*** for 2 h at 32°C. Bone marrow-derived T-cell progenitors used for viral transduction were cultured for 6-7 days according to conditions described above, disaggregated, filtered through a 40-μm nylon mesh, and 10^6^ cells were transferred onto each RetroNectin/DL1-coated virus-bound well supplemented with 5 ng/ml SCF (Peprotech), 5 ng/ml Flt3-L, and 5 ng/ml IL-7. *Zbtb16* cDNA (NM_006006, Twist Bioscience) was cloned into a MSCV-IRES-mCherry backbone via Gibson Assembly (Kueh et al., 2013). Retroviral particles were generated using the Pheonix-Eco 293T cell line and viral supernatant was collected 48 h following transfection. Bone marrow progenitors were infected following a RetroNectin (Takara Bio) transduction protocol. Briefly, a 24-well plate was coated with RetroNectin at 12.5 μg/ml and stored overnight at 4°C. The next day, 2 ml of fresh viral supernatant was spun onto the well plate at 3000 ***g*** for 2 h. Subsequently, 2 million progenitor cells were seeded on top of the viral plate and spun at 800 ***g*** for 30 min before resuspending and transferring to OP9-DL1 stromal cells.

## Supplementary Material

10.1242/develop.203016_sup1 Supplementary information

Table S1. Top 100 genes in each MIRA gene topic.
