# Supplementary material for: A timed epigenetic switch balances T and ILC lineage proportions in the thymus
Source: Development. 2024 Dec 10;151(23):dev203016. doi: 10.1242/dev.203016 (PMC11664168; doi:10.1242/dev.203016)
Supplement: Supplementary information [file develop-151-203016-s1.pdf]

A

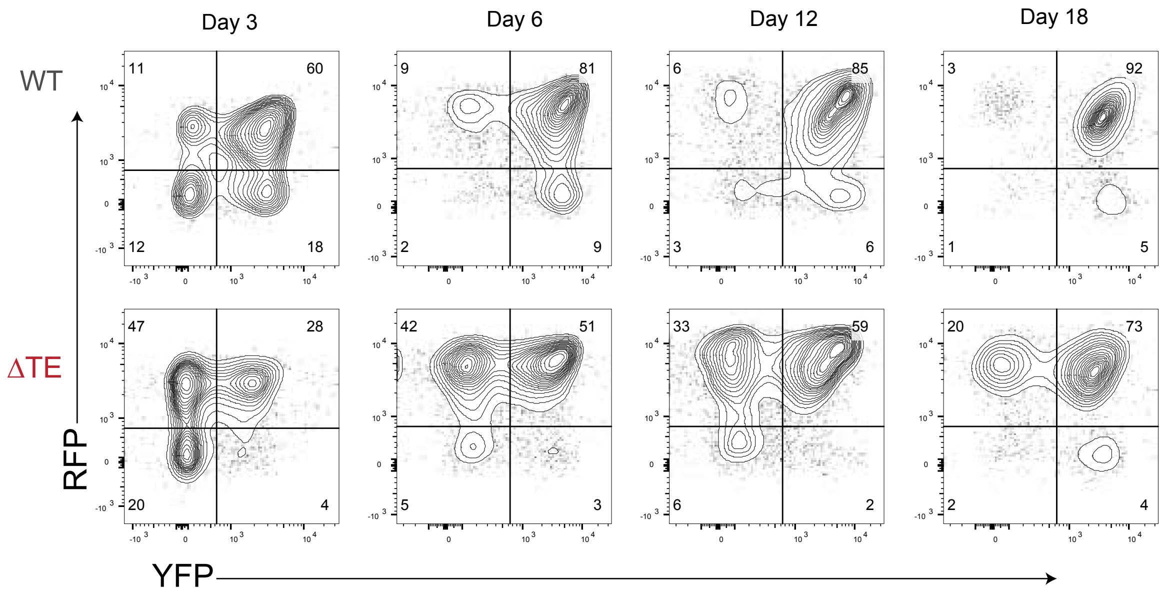

Fig. S1. Timing enhancer regulates *Bcl11b* timing in cis. (A) Representative contour plots for Bcl11b-RFP and -YFP levels of purified DN2a progenitors re-cultured on OP9-DL1 cells.

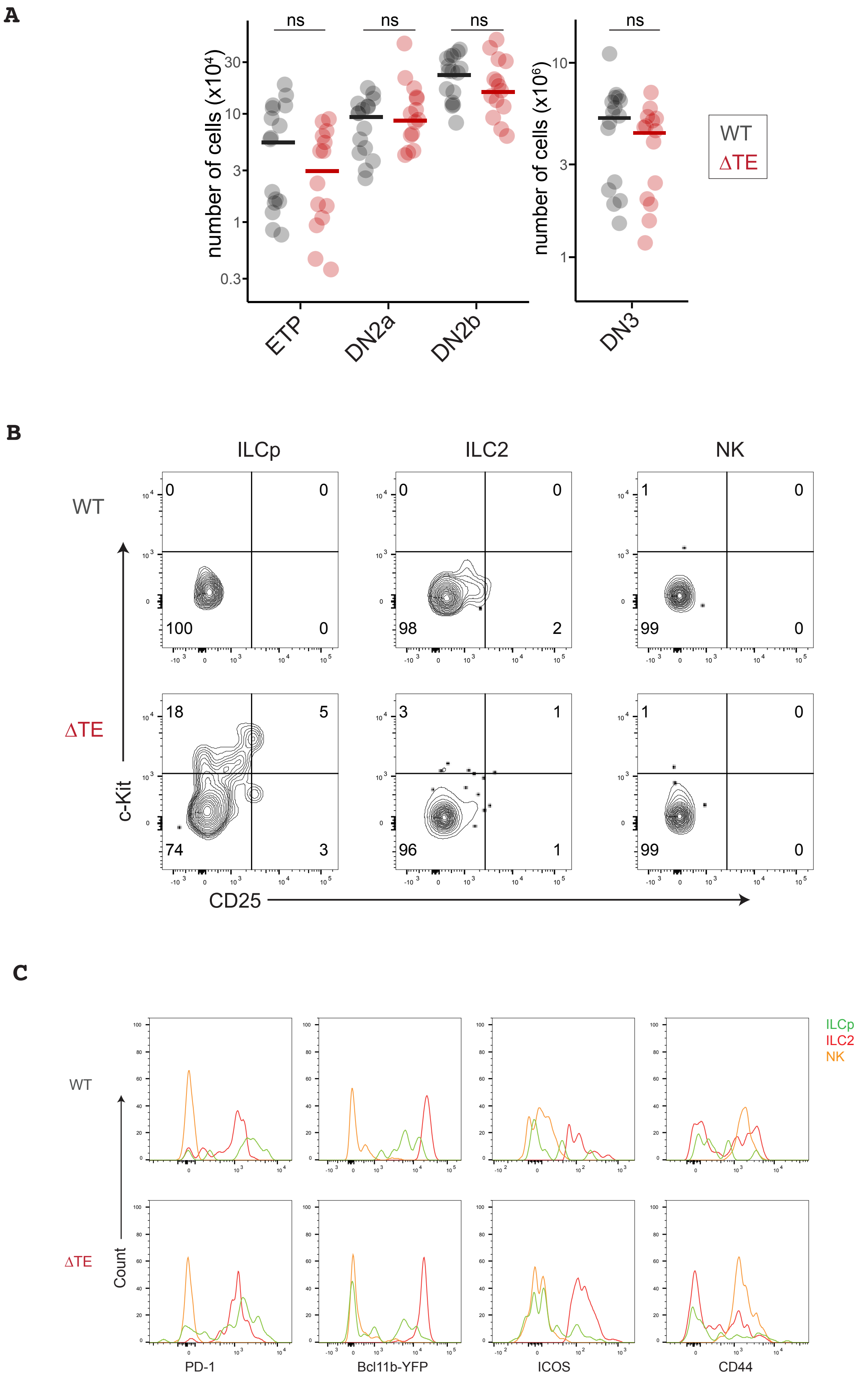

Fig. S2. Immunophenotyping of thymic progenitor populations. (A) Quantification of thymocyte population frequencies and total cell numbers (unpaired Wilcoxon rank sum test (two-tailed), *p < 0.05, **p < 0.01, n = 16 separate WT mice and n = 14 separate ΔTE mice for DN populations). (B) Representative contour plots of thymic ILC subsets. (C) Representative histograms for ILC related markers.

Development • Supplementary information

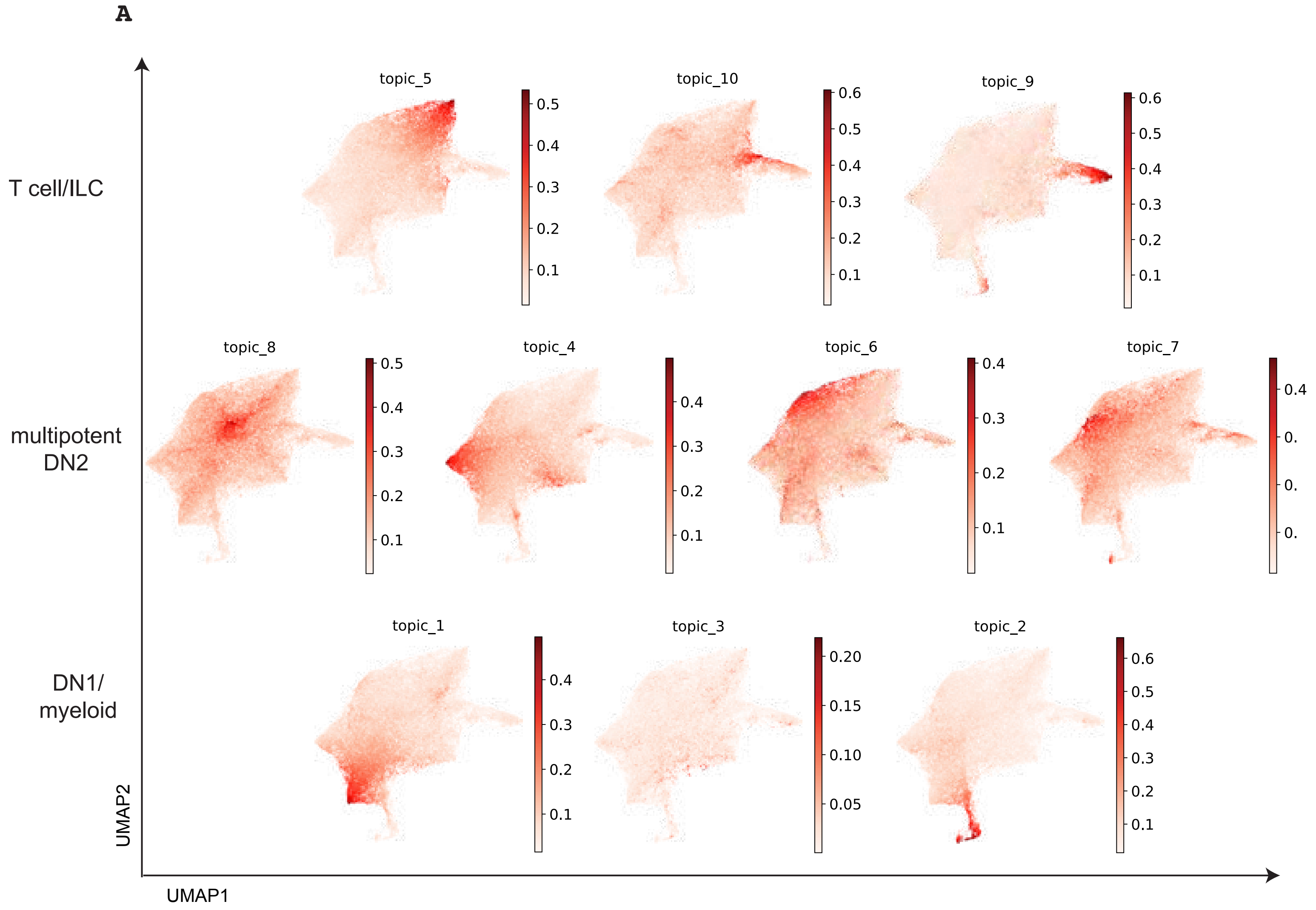

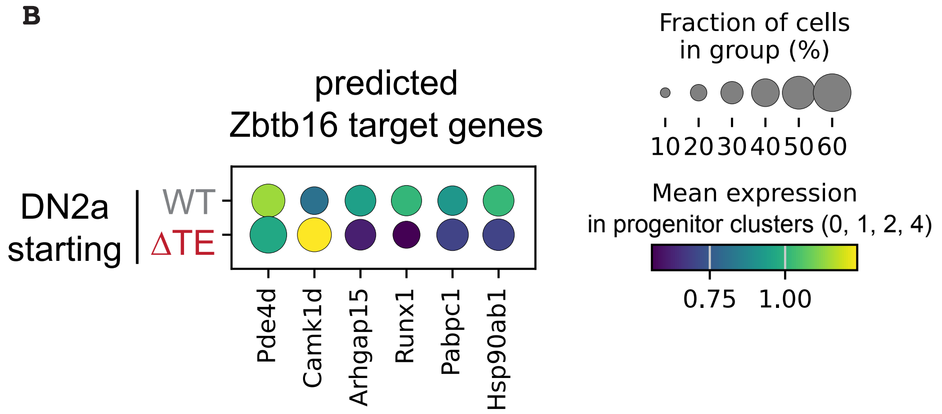

Fig. S3. sci-RNA-seq gene topics and Zbtb16 target gene expression. (A) UMAP repre-sentation of all cells colored by gene topic expression. (B) Normalized expression levels of selected top CellOracle predicted Zbtb16 target genes across in DN2a-starting cells in the progenitor clusters.

Table S1. Top 100 genes in each MIRA gene topic.

Available for download at
https://journals.biologists.com/dev/article-lookup/doi/10.1242/dev.203016#supplementary-data

Development • Supplementary information

Table S2. Antibodies used in this manuscript.

Antibody	Source	Identifier	Dilution
CD25 BV510	Biolegend	Cat# 102042; RRID:AB_2562270	1:30
C44 PerCP-Cy5.5	Invitrogen	Cat# 45-0441-82; RRID:AB_925746	1:300
CD117 (cKit) eFlour-450	eBioscience	Cat# 48-1171-82; RRID:AB_2574037	1:200
CD19-biotin	eBioscience	Cat#13-0193-85; RRID:AB_657658	1:100
CD11b-biotin	eBioscience	Cat#13-0112-86; RRID:AB_466361	1:100
CD11c-biotin	eBioscience	Cat#13-0114-85; RRID:AB_466364	1:100
NK1.1-biotin	eBioscience	Cat#13-5941-85; RRID:AB_466805	1:100
Ter119-biotin	eBioscience	Cat#13-5921-85; RRID:AB_466798	1:100
CD3e-biotin	eBioscience	Cat#13-0031-85; RRID:AB_466320	1:100
Gr-1-biotin	eBioscience	Cat#13-5931-86; RRID:AB_466802	1:100
B220-biotin	eBioscience	Cat#13-0452-85; RRID:AB_466450	1:100
Streptavidin BV711	Biolegend	Cat#405241	1:300
CD4 BV510	Biolegend	Cat# 100449;RRID:AB_2564587	1:300
CD8 APC	eBioscience	Cat# 17-0081-82; RRID:AB_469335	1:300
CD122 PE	Biolegend	Cat# 123209; RRID:AB_940615	1:200
TCR-beta PE/Cy-7	Biolegend	Cat# 109221; RRID:AB_893627	1:150
CD49b (DX5) PE/Dazzle-594	Biolegend	Cat# 108923; RRID:AB_2565270	1:200
ICOS APC/FIRE-750	Biolegend	Cat# 313535; RRID:AB_2632922	1:100
PD1 APC	Invitrogen	Cat# 17-9981-82; RRID:AB_10852564	1:40
PLZF PE-Cy7	Biolegend	Cat# 145805; RRID:AB_2566164	1:400

Development • Supplementary information
